# *Enterococcus faecalis* biofilm removal efficacy, cytotoxicity and alkaline phosphatase activity on stem cells after the application of conventional medicaments and novel hydrogels used in regenerative endodontics

**DOI:** 10.1186/s12903-026-08217-6

**Published:** 2026-04-02

**Authors:** Adem Öztürk, Tuğçe Duran, Ayce Unverdi Eldeniz

**Affiliations:** 1 Department of Endodontics, Faculty of Dentistry, Selcuk University, Konya, Turkey; 2 Department of Medical Genetics, Faculty of Medicine, KTO Karatay University, Konya, Turkey

**Keywords:** *Enterococcus faecalis*, hydrogel, intracanal medicaments, regenerative endodontics, stem cells

## Abstract

**Background:**

To compare the antibacterial activity of conventional intracanal medicaments used in regenerative endodontic treatment (TAP, DAP, Ca(OH)₂, CHX gel) with novel hydrogel-based formulations (chitosan, CHX, hyaluronic acid, metronidazole, and diclofenac hydrogels) against *Enterococcus faecalis* biofilms, and to assess their cytotoxic effects on dental pulp stem cells (DPSCs).

**Methods:**

Antibacterial activity was evaluated using an *E. faecalis* biofilm model in single-rooted incisors. For cytotoxicity testing, dentine discs were treated with the medicaments and seeded with DPSCs. Cell viability was assessed using the WST-1 assay, and mineralization-related responses were evaluated by alkaline phosphatase (ALP) activity. Data were analyzed using the Kruskal–Wallis test with Bonferroni-adjusted post-hoc comparisons (*p* < 0.05).

**Results:**

TAP, DAP, diclofenac hydrogel, and CHX hydrogel completely eliminated the *E. faecalis* biofilm in the CFU analysis, whereas CHX gel reduced the biofilm but did not achieve full eradication. In contrast, Ca(OH)₂, chitosan, HA, and MTR hydrogels exhibited limited antibacterial effects. Ca(OH)₂ and chitosan hydrogel preserved the highest levels of cell viability, while DAP, TAP, HA, MTR, and diclofenac hydrogels caused moderate reductions; CHX gel and CHX hydrogel showed the lowest survival. ALP activity was maintained in the Ca(OH)₂, chitosan, HA, MTR, and diclofenac hydrogels but decreased in the CHX based groups (all relevant comparisons, *p* < 0.05).

**Conclusions:**

Hydrogel-based systems demonstrated a favorable balance between biofilm control and stem-cell biocompatibility. The diclofenac-loaded hydrogel achieved TAP/DAP-level biofilm reduction while preserving cell viability, supporting its potential as an antibiotic-free disinfection alternative. CHX hydrogel showed higher antibacterial performance than CHX gel, whereas hyaluronic acid hydrogel maintained high biocompatibility and demonstrated applicability as a biofunctional carrier system.

## Introduction

 Regenerative endodontic procedures (REPs) are biologically based treatment methods aimed at repairing or regenerating damaged tissues in dentine, root structures, and the pulp-dentine complex through biological processes [1]. Regenerative endodontic procedures are widely applied, particularly in the treatment of immature teeth with pulp necrosis and apical periodontitis. They support apical closure and continued root development by promoting symptom resolution, improvement in periapical radiolucency, thickening of root canal walls, and the restoration of pulp connective tissue [2–4]. However, studies have shown that bacteria remaining in the root canal system cannot be completely eliminated, and these bacteria may negatively affect the regeneration process. The persistence of resistant bacteria in root canals can lead to continued infection, hinder tissue healing, and limit the success of REPs, making it more difficult to achieve the desired biological response [5, 6]. In line with this, recent evidence suggests that the overall clinical effectiveness of revitalization procedures for apical periodontitis remains uncertain, with limited high-quality long-term data available [7].

In REPs, minimal shaping of root canal walls makes microbial biofilm elimination highly dependent on the effectiveness of irrigation solutions and intracanal medicaments. These solutions and medicaments should not only eliminate bacteria but also preserve stem cell viability and create a favorable environment that supports the biological functions of the dentine matrix [8, 9]. In this context, the most commonly used intracanal medicaments in REPs include calcium hydroxide (Ca(OH)₂) and antibiotic combinations. These combinations can be applied as triple antibiotic paste (TAP) containing equal proportions of ciprofloxacin, metronidazole, and minocycline; double antibiotic paste (DAP) without minocycline; or modified TAP formulations [10–13]. The American Association of Endodontists (AAE) recommends the use of intracanal medicaments as a supplementary disinfection step in REPs. In this regard, AAE has specifically recommended the use of Ca(OH)₂ or antibiotic-based pastes, particularly TAP and DAP, for disinfection purposes during regenerative procedures [14].

Calcium hydroxide is classified as a strong base with a high pH (approximately 12.5–12.8). Its mechanism of action occurs through the dissociation of Ca²⁺ and OH⁻ ions, which exert biological effects on vital tissues. Hydroxyl ions exhibit antimicrobial activity against microorganisms through protein denaturation, DNA damage, and cytoplasmic membrane disruption [15]. Clinically used concentrations of Ca(OH)₂ have been shown to be non-toxic to stem cells from the apical papilla (SCAP) and dental pulp stem cells (DPSC). Ca(OH)₂ has been reported to support SCAP and DPSC proliferation while maintaining biocompatibility when interacting with the dentine surface, positively influencing regenerative processes [15–17]. However, it has been reported that Ca(OH)₂ has limited effectiveness against *E. faecalis* biofilms, which may negatively impact treatment outcomes [18, 19].

Although antibiotic pastes exhibit strong antibacterial effects, they may trigger prolonged inflammatory responses [16, 20]. Therefore, to minimize their adverse effects on stem cell viability and proliferation, antibiotics are recommended to be used at low concentrations (1–5 mg/mL) [14]. However, while lower concentrations of antibiotic pastes are better tolerated by cells, their effectiveness against intracanal bacterial biofilms is reduced [21, 22]. The use of antibiotics may lead to the development of antibiotic-resistant microorganisms in biofilms and has been associated with reduced dentine strength, coronal discoloration, and adverse effects on host cells [20, 23, 24]. Despite these biological concerns, antibiotic pastes continue to be widely used in clinical practice. A multinational survey reported that 41.7% of clinicians employ antibiotic pastes in regenerative endodontic procedures, reflecting their ongoing clinical relevance [25]. More recent data further demonstrate that the use of regenerative endodontic procedures and antibiotic pastes varies considerably across countries and specialties, highlighting the lack of reliable standardized clinical protocols [26].

Since current disinfection methods do not provide the desired level of effectiveness, new alternatives for root canal disinfection in REPs need to be explored [27, 28]. Chitosan has been increasingly investigated in regenerative endodontics as a biocompatible polymeric scaffold capable of functioning as a local drug-delivery system [29, 30]. Hydrogel and nanoparticle formulations have been developed to enhance intracanal retention and enable controlled release of antimicrobial agents [29, 30]. However, when used alone, chitosan may exhibit limited antibiofilm activity against *E. faecalis*, and its antimicrobial performance is often dependent on the incorporated agents [31, 32]. Moreover, as with other gel-based intracanal materials, its interaction with dentine surfaces should be considered in regenerative procedures [33].

The aim of this in vitro study is to compare the antibacterial activities of conventional intracanal medicaments used in regenerative endodontic treatment, such as TAP, DAP, Ca(OH)₂, and CHX gel, with novel intracanal medicaments such as chitosan hydrogel, CHX hydrogel, hyaluronic acid hydrogel, metronidazole hydrogel, and diclofenac hydrogel, while also evaluating their effects on the viability of dental pulp stem cells (DPSCs).

## Materials and methods

### Part 1: Sample preparation for antibacterial activity evaluation

In the first phase of the study, single-rooted, non-carious and non-curved extracted human maxillary central incisors (*n* = 121) were used to evaluate the antibacterial efficacy of intracanal medicaments against *E. faecalis*. As part of the sample preparation, the coronal portion was removed under water cooling, resulting in a 15-mm root segment. The root canals were then prepared up to size F4 using the ProTaper Universal system (Dentsply Maillefer, Ballaigues, Switzerland). To ensure sterilization, the specimens were placed in an ultrasonic bath containing 17% EDTA for 3 min, followed by immersion in 5.25% NaOCl for another 3 min. Subsequently, all samples were autoclaved at 121 °C. To verify sterility, each sample was placed in Eppendorf tubes containing sterile BHI broth (Merck KGaA, Darmstadt, Germany) and incubated at 37 °C for 48 h, and the medium was visually inspected daily for turbidity. The teeth were then randomly allocated into 11 groups (*n* = 11 per group); of these, 10 specimens in each group were used for CFU analysis, whereas one specimen per group was reserved for SEM evaluation as a qualitative representative sample.

### Bacterial culture preparation and root canal contamination

The *Enterococcus faecalis* (A197A) strain was reactivated from frozen stock cultures under aerobic conditions at 37 °C in Brain Heart Infusion (BHI) broth (Merck KGaA, Darmstadt, Germany) and cultivated until the late logarithmic or early stationary phase. The bacterial suspension was then centrifuged, and the pellet was resuspended in fresh BHI broth. The bacterial concentration was adjusted spectrophotometrically to an optical density of OD600 = 0.6. Subsequently, the root specimens were inoculated with 10 mL of bacterial suspension, transferred into 15 mL tubes, and incubated in 5 mL of BHI broth. To maintain bacterial viability, the culture medium was refreshed every three days. All specimens were incubated at 37 °C for 21 days to allow bacterial growth.

### Preparation of intracanal medications

A suspension was prepared by dissolving 1 g of chitosan and 0.1 g of the active agent (Diclofenac Potassium, Metronidazole, Hyaluronic acid, or Chlorhexidine) in 60 mL of water containing 1% (v/v) acetic acid. Subsequently, 0.36 g (0.27 mmol) of DIC (N, N-diisopropylcarbodiimide) was added to the mixture. The solution was stirred at room temperature under a nitrogen atmosphere for 2 h, followed by heating at 70 °C for 20 h. The hydrogel consistency was concentrated to obtain a ready-to-use formulation [34]. In addition, a chitosan-based hydrogel prepared using the same formulation protocol but without incorporation of any active agent was included as a vehicle control to allow discrimination between the effect of the carrier matrix and that of the incorporated medicaments. Double and triple antibiotic pastes were also prepared by removing the tablet coatings of metronidazole 500 mg (Nidazol; İbrahim Etem Ulagay, Istanbul, Turkey), ciprofloxacin 500 mg (Cipro; Biofarma, Istanbul, Turkey), and doxycycline 100 mg (Tetradox; Teva, Istanbul, Turkey), pulverizing the tablets (DAP: 1:1; TAP: 1:1:1), and mixing the resulting powders with sterile distilled water to obtain a 2.5 mg/mL paste formulation.

### Intracanal medication application

After the incubation period, the external surfaces of the specimens were wiped with 70% ethanol to remove residual bacteria, and the internal surfaces of the root canals were dried using sterile paper points. The root canals of all specimens in each group were then filled with the assigned medicaments. TAP, DAP, chitosan hydrogel, CHX hydrogel, hyaluronic acid hydrogel, metronidazole hydrogel, and diclofenac hydrogel were applied using sterile syringes, whereas Ca(OH)₂ and CHX gel were delivered with their respective applicator systems. Untreated infected canals served as the positive control, and non-infected, untreated canals served as the negative control. After medication placement, the canal orifices were sealed with Cavit (3 M ESPE, St. Paul, MN), and the specimens were incubated at 37 °C for seven days. Of the 11 specimens in each group, 10 were used for CFU analysis, while one specimen was reserved for SEM evaluation.

### CFU analysis

After the incubation period, the apical 3 mm of 10 specimens per group was removed using a disc, and the remaining medicaments in the root canals were eliminated by rinsing with 5 mL of sterile distilled water. After drying the canals with sterile paper points, the teeth were positioned over sterile glass vials containing 2 mL of phosphate-buffered saline (PBS, Sigma-Aldrich, Steinheim, Germany), and dentine shavings were collected directly into the vials using Gates-Glidden drills (#3, #4, and #5) in sequence. Aseptic conditions were maintained throughout the procedure, ensuring that the burs did not contact the external surfaces of the root segments. The collected dentine samples were vortex-mixed for 30 s to obtain a homogeneous suspension. The bacterial suspension was then serially diluted to 10⁻¹, 10⁻², 10⁻³, and 10⁻⁴, and 25 µL from each dilution was plated onto Tryptic Soy Agar (TSA) plates. After incubation at 37 °C for 48 h in an inverted position, bacterial colonies were counted, and the colony-forming units (CFU/mL) were calculated and log10-transformed for statistical analysis.

### Preparation of root specimens for SEM analysis

After the incubation period was completed, a randomly selected specimen from each group was subjected to scanning electron microscopy (SEM) analysis to evaluate the efficacy of the medicaments against *E. faecalis* biofilm and their ability to occlude dentinal tubules. The specimens were prepared by creating a separation line using a disc along the buccolingual direction of the root surface without contacting the canal walls. The samples were then split into two halves, mesial and distal, using a spatula placed along the separation line. To preserve structural integrity, the specimens were fixed in 2% glutaraldehyde solution and incubated in an oven at 37 °C for 48 h to ensure complete drying before SEM analysis. Once adequate dryness was achieved, the specimens were coated with gold and examined under a scanning electron microscope (ZEISS EVO LS 10, Germany) at various magnifications.

### Part 2: Primary culture of human dental pulp stem cells (DPSC)

For the primary culture of human dental pulp-derived stem cells (DPSC), high-glucose, bicarbonate-buffered, and phenol red indicator-containing DMEM (Dulbecco’s Modified Eagle Medium, Biowest, France) was used. The medium was freshly prepared by supplementing it with 10% heat-inactivated fetal bovine serum (FBS, Capricorn Scientific, Germany), 1% penicillin/streptomycin (Capricorn Scientific, Germany), and 3% antibiotic/antimycotic (Gibco, USA). When the DPSCs reached 80–90% confluency on the flask surface, passaging was performed.

After the removal of the culture medium, 0.25% trypsin-EDTA (Gibco, USA) was added to detach the cells from the surface, followed by incubation at 37 °C in a 5% CO₂ incubator for 10 min. To neutralize the trypsin activity, twice the volume of fresh culture medium was added, and the suspension was centrifuged at 2000 rpm for 5 min to collect the cells. After centrifugation, the supernatant was discarded, and the cell pellet was resuspended in fresh culture medium and seeded into new flasks at a density of approximately 1–4 × 10⁴ cells/cm². The flasks were maintained in a humidified incubator at 37 °C with 5% CO₂ to continue cell culture.

### Preparation of dentine discs and medication application

In the second phase of the study, a total of 100 dentine discs, each measuring 1 mm in thickness and 5–6 mm in diameter, were prepared and placed into well plates. Ten different groups were formed, each consisting of 10 discs, and arranged for four different analyses. Prior to medicament application, the discs were treated sequentially with 2 mL of 5.25% sodium hypochlorite, 2 mL of physiological saline, and 2 mL of 17% EDTA solution for 5 min. The EDTA treatment was performed to remove the smear layer and standardize the dentine surface prior to medicament application. This was followed by three rinses with 2 mL of PBS, and the discs were sterilized in an autoclave at 121 °C for 20 min. After sterilization, the surface of each disc, except for those in the control groups, was coated with the designated medicament. Finally, the discs were placed into 24-well plates and incubated for seven days in a cell culture incubator at 37 °C under 5% CO₂.

### Cell viability assessment

After the incubation period was completed, DPSCs (4 × 10⁴ cells/disc) were seeded onto medicament-treated dentine discs and cultured for seven days. At the end of the incubation period, cell viability was assessed using the WST-1 Cell Viability Assay Kit (Cayman Chemical, USA). Discs containing DPSCs without medicament application served as the positive control group, whereas discs containing neither medicament nor DPSCs were designated as the negative control group. After removal of the culture medium, WST-1 reagent was added and the samples were incubated for four hours. The resulting supernatant was transferred into 96-well plates in triplicate, and absorbance was measured at 450 nm using an Epoch BioTek (USA) microplate reader. The measurements were repeated three times to confirm the results.

### Alkaline phosphatase activity

After one week of culture, DPSCs (4 × 10⁴ cells/disc) were assessed for alkaline phosphatase (ALP) activity using the ALP Assay Kit (Elabscience, China) (*n* = 3/group). Prior to testing, the kit, which had been stored at -20 °C, was equilibrated to room temperature according to the manufacturer’s instructions, and a working solution was prepared by mixing 3 mL of buffer solution with one bottle of substrate, followed by storage in the dark at -20 °C for 24 h. After the storage period, DPSCs were lysed using a sonicator, and the supernatant was obtained by centrifugation. Fifty microliters (50 µL) of the supernatant from each sample was transferred into 96-well microplates, followed by the addition of 50 µL of substrate working solution to each well. The plates were then incubated at 37 °C for 10 min, after which 100 µL of stop solution was added to each well. Absorbance was measured at 405 nm using an Epoch BioTek (USA) microplate reader, and all measurements were performed in triplicate to ensure accuracy.

### Confocal laser scanning microscopic analysis

The morphological evaluation of DPSCs was performed using the Viability/Cytotoxicity Assay Kit. According to the manufacturer’s instructions, 5 µL of SYTOX™ Deep Red Nucleic Acid Stain (660/682 nm, marking dead cells in red) was mixed with 10 mL of medium and vortexed, followed by the addition of 2.5 µL of Calcein, AM (494/517 nm, marking live cells in green) and further vortexing to prepare the working solution for cell staining. The culture medium in the wells of the microplate was removed, and the working solution was added to the discs. The staining process was carried out by incubating the samples for 30 min in an incubator, ensuring that all procedures were performed in a dark environment to protect the light-sensitive dyes. The images of three samples from each group were recorded.

### Preparation of dentine discs for SEM analysis

Scanning electron microscopy (SEM) analysis was performed to examine stem cell adhesion. One disc from each group was carefully washed three times with PBS and then fixed with 2% glutaraldehyde for 30 min to preserve cellular structures. After fixation, a gradual dehydration process was carried out using ethanol series of increasing concentrations to facilitate drying. In this process, the discs were sequentially immersed in 20%, 50%, 70%, and 90% ethanol solutions for 2 h each. In the final stage, the samples were incubated in 99% ethanol for 24 h, completing the drying process. Once adequate dryness was achieved, the specimens were coated with gold and examined under a scanning electron microscope (ZEISS EVO LS 10, Germany) at various magnifications.

### Statistical analysis

Quantitative variables were expressed as mean, standard deviation, median, minimum, and maximum values. CFU counts were calculated based on dilution factors and plating volume and were subsequently log10-transformed for reporting purposes, as commonly applied in endodontic microbiological studies [35]. Samples demonstrating complete bacterial eradication were retained as zero values in the analysis. Normality was assessed using the Kolmogorov–Smirnov and Shapiro–Wilk tests. As the data were not normally distributed and included zero values, between-group comparisons were performed using the rank-based Kruskal–Wallis H test, followed by Bonferroni-adjusted post-hoc analyses where appropriate. Statistical significance was set at *p* < 0.05. All analyses were conducted using R (v4.1.2) and IBM SPSS Statistics (Version 25.0).

Sample size was determined using G*Power (effect size = 0.45; power = 0.80; α = 0.05), resulting in 10 specimens per group for CFU analysis. Biological assays were performed with *n* = 3 per group and analyzed in triplicate.

## Results

### Part 1: Antibacterial effects of intracanal medicaments

**Fig. 1:**
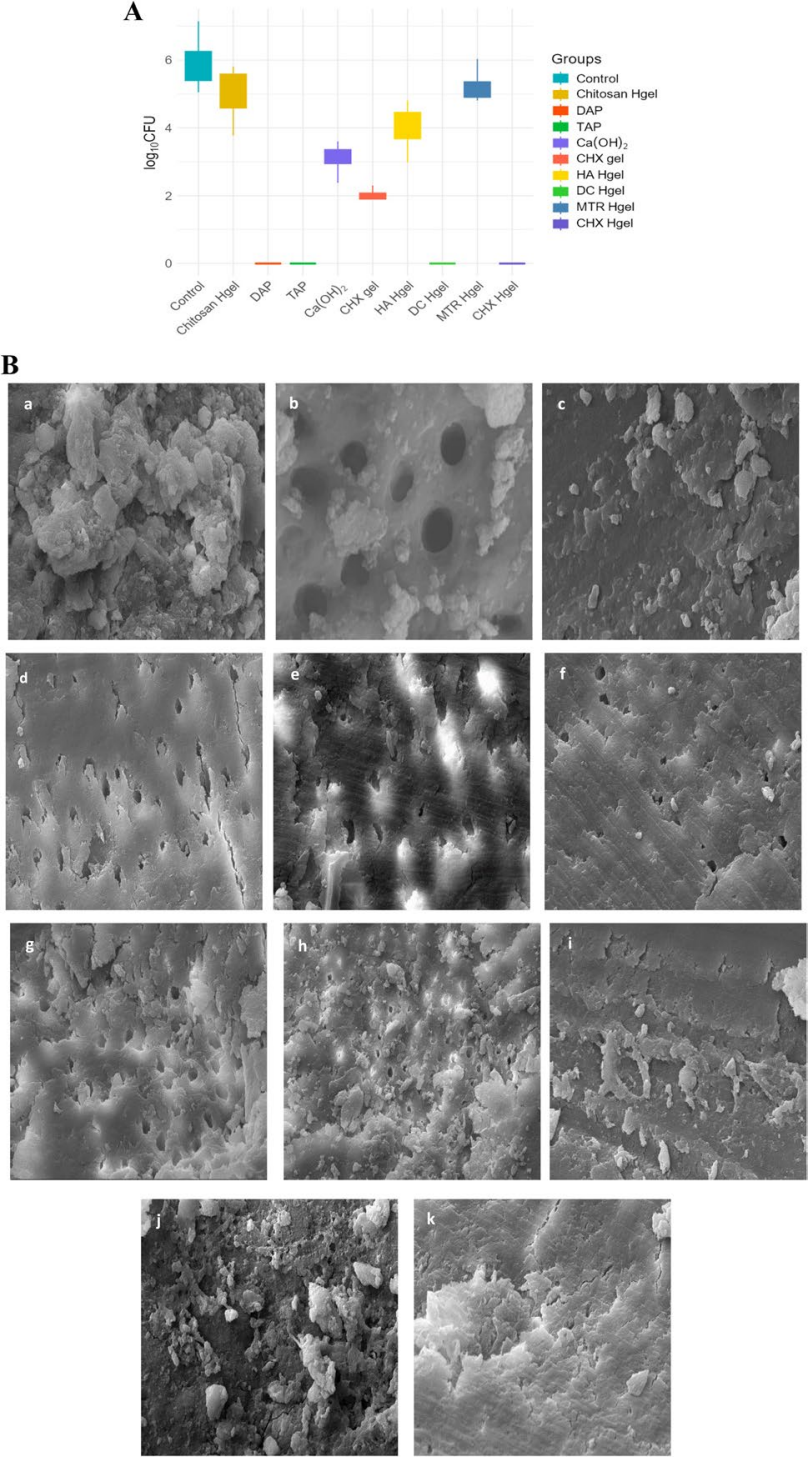
(**A**)* Enterococcus faecalis* bacterial load (Log₁₀ CFU) after intracanal medication in the study groups (*n* = 10/group). (**B**) After a three-week infection period, the biofilm structure of the E. faecalis A197A strain was observed in the positive control group. In the negative control group, open dentinal tubules were observed without the presence of Enterococcus species. After completion of the infection period, the designated medicaments were applied to the experimental groups for one week, and the resulting surfaces were examined using scanning electron microscopy (SEM) (original magnification, ×15,000). Panel labels: a Positive control; b Negative control; c Chitosan Hgel; d DAP; e TAP; f Ca(OH)₂; g CHX gel; h HA Hgel; i DC Hgel; j MTR Hgel; k CHX Hgel

The antibacterial effects of the intracanal medicaments against *E. faecalis* biofilm were statistically evaluated. Table 1 presents the mean (SD), median, minimum, and maximum Log_10_ CFU values for all study groups. The mean Log_10_ CFU value in the positive control group was 5.88 ± 0.21, whereas no bacterial growth was observed in the negative control group. Compared with the positive control, most medicament groups showed a significant reduction in bacterial load. The greatest antibacterial effect was observed in the DAP, TAP, diclofenac-hydrogel, and CHX Hgel groups, in which no bacterial growth was detected (CFU = 0). Although the CHX gel group also showed a significant decrease in bacterial load, lower antibacterial activity was observed in the Ca(OH)₂, MTR-hydrogel, and chitosan-hydrogel groups (Fig. 1A). SEM analysis confirmed the presence of dense biofilm structures in the infected control group and the absence of bacteria with open dentinal tubules in the negative control group. In the medicament groups, SEM images qualitatively supported the CFU findings and illustrated surface changes associated with biofilm removal (Fig. 1B).

**Table 1:** Comparison of measurement values obtained after the application of intracanal medicaments to *E. faecalis*-infected incisor root specimens

Groups	Mean (SD)	Median (Log CFUs)	Minimum	Maximum	*p*-value
Control	5.88 (0.21)	5.82^a^	5.05	7.15	< 0.001
Chitosan Hgel	4.93 (0.23)	4.98^bj^	3.78	5.81	
DAP	0.0 (0.0)	0.0^cdhk^	0.0	0.0	
TAP	0.0 (0.0)	0.0^dhk^	0.0	0.0	
Ca(OH)_2_	3.11 (0.11)	3.14^e^	2.38	3.6	
CHX gel	1.98 (0.08)	1.99^f^	1.6	2.3	
HA Hgel	3.89 (0.2)	3.75^g^	2.98	4.81	
DC Hgel	0.0 (0.0)	0.0^hk^	0.0	0.0	
MTR Hgel	5.12 (0.15)	5.06^j^	4.2	6.03	
CHX Hgel	0.0 (0.0)	0.0^k^	0.0	0.0	

Differences between groups were analyzed using the Kruskal–Wallis test (*p* < 0.05), followed by Bonferroni-adjusted post hoc multiple comparisons. Superscript letters represent the results of pairwise comparisons. Groups sharing at least one common superscript letter show no statistically significant difference, whereas groups with no shared letters show a statistically significant difference (*p* < 0.05)

*Abbreviations*: Chitosan Hgel, Chitosan Hydrogel; *DAP*, Double Antibiotic Paste; *TAP*, Triple Antibiotic Paste; Ca(OH)₂, Calcium Hydroxide Paste; *CHX gel*, Chlorhexidine Gel; *HA Hgel*, Hyaluronic Acid Hydrogel; *DC Hgel*, Diclofenac Hydrogel; *MTR Hgel*, Metronidazole Hydrogel; *CHX Hgel*, Chlorhexidine Hydrogel

### Part 2: Cell viability, morphology, and ALP activity

**Fig. 2:**
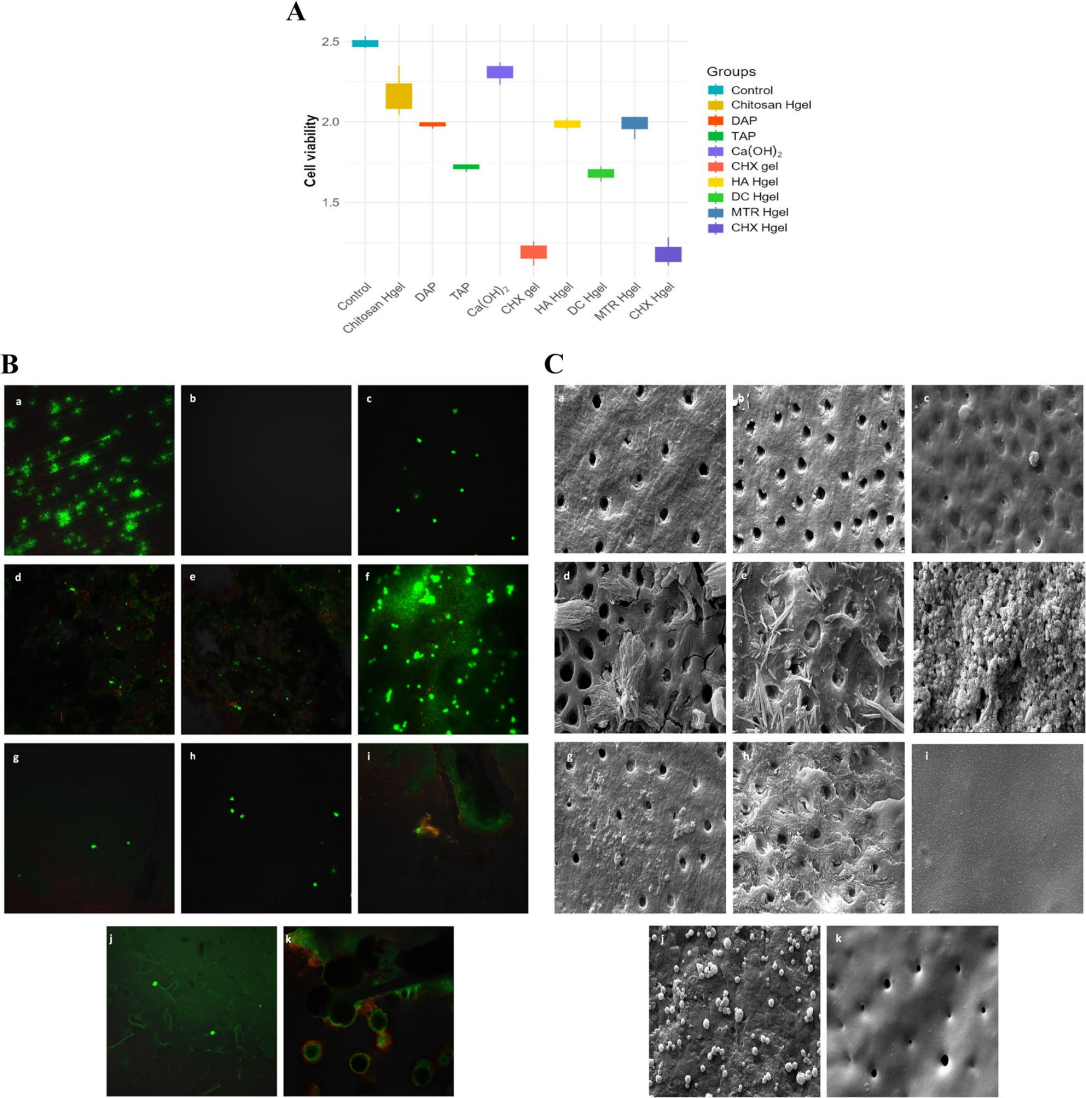
**A** Cell viability of dental pulp stem cells (DPSCs) exposed to the medicaments was evaluated after 7 days using the WST-1 assay (*n* = 3 per group, analyzed in triplicate). **B** Fluorescence microscopy images show live (green) and dead (red) DPSCs cultured on the medicament-treated dentine surface. DPSCs cultured on the untreated dentine surface were used as the positive control (*n* = 3/group). Panel labels: a Positive control; b Negative control; c Chitosan Hgel; d DAP; e TAP; f Ca(OH)₂; g CHX gel; h HA Hgel; i DC Hgel; j MTR Hgel; k CHX Hgel. **C** Scanning electron microscopy (SEM) images of DPSCs showing variations in cell adhesion, spreading, and surface morphology depending on the applied medicaments (original magnification, ×20,000). Panel labels: a Positive control; b Negative control; c Chitosan Hgel; d DAP; e TAP; f Ca(OH)₂; g CHX gel; h HA Hgel; i DC Hgel; j MTR Hgel; k CHX Hgel

In this study, the cytotoxic effects and alkaline phosphatase (ALP) activity of intracanal medicaments tested on dental pulp stem cells (DPSCs) cultured on dentine discs were statistically analyzed. Table 2 presents the mean (SD), median, minimum, and maximum values of cell viability for each experimental group. According to the WST-1 assay results, the highest cell viability was observed in the positive control group, in which the cells were cultured on untreated dentine surfaces without medicament application. No significant difference was found between the Ca(OH)₂ and chitosan-hydrogel-treated dentine surfaces and the positive control group. In contrast, the lowest DPSC viability values were recorded in the chlorhexidine gel (CHX gel) and chlorhexidine hydrogel (CHX Hgel) groups (Fig. 2A).

**Table 2:** Comparison of cell viability values obtained using the WST-1 assay after 7 days of cell seeding on medicament-treated dentine discs

Groups	Mean (SD)	Median (Cell viability)	Minimum	Maximum	*p*-value
Control	2.49 (0.022)	2.48^a^	2.46	2.53	0.001
Chitosan Hgel	2.17 (0.092)	2.12^ag^	2.04	2.35	
DAP	1.98 (0.012)	1.99^bceg^	1.96	1.99	
TAP	1.72 (0.015)	1.73^cef^	1.69	1.74	
Ca(OH)_2_	2.31 (0.041)	2.32^a^	2.23	2.37	
CHX gel	1.19 (0.044)	1.2^dfh^	1.11	1.26	
HA Hgel	1.99 (0.023)	1.99^eg^	1.95	2.03	
DC Hgel	1.68 (0.028)	1.68^fh^	1.63	1.72	
MTR Hgel	1.98 (0.046)	2.02^g^	1.89	2.03	
CHX Hgel	1.18 (0.052)	1.16^h^	1.11	1.28	

Differences between groups were analyzed using the Kruskal–Wallis test (*p* < 0.05), followed by Bonferroni-adjusted post hoc multiple comparisons. Superscript letters represent the results of pairwise comparisons. Groups sharing at least one common superscript letter show no statistically significant difference, whereas groups with no shared letters show a statistically significant difference (*p* < 0.05)

*Abbreviations*: Chitosan Hgel, Chitosan Hydrogel; *DAP*, Double Antibiotic Paste; *TAP*, Triple Antibiotic Paste; *Ca(OH)₂*, Calcium Hydroxide Paste; *CHX gel*, Chlorhexidine Gel; *HA Hgel*, Hyaluronic Acid Hydrogel; *DC Hgel*, Diclofenac Hydrogel; *MTR Hgel*, Metronidazole Hydrogel; *CHX Hgel*, Chlorhexidine Hydrogel

**Fig. 3:**
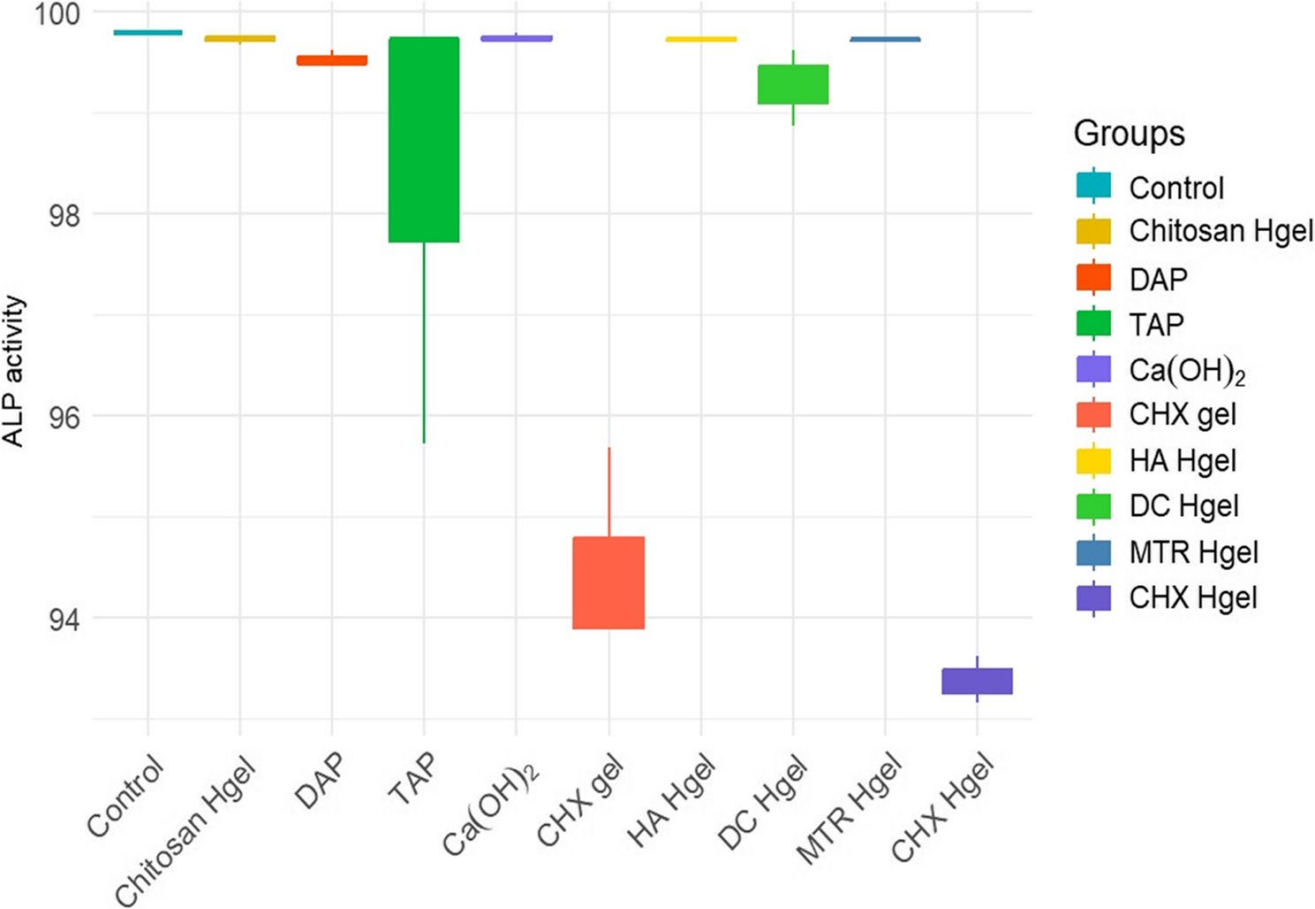
ALP activity of dental pulp stem cells (DPSCs) after 7-day exposure to the medicaments, evaluated using the ALP assay (*n* = 3 per group, analyzed in triplicate)

Table 3 presents the measurement values obtained for ALP activity in the experimental groups. ALP activity was analyzed as an indicator of the early osteogenic potential of DPSCs following exposure to the different medicaments. The highest ALP activity was observed in the control group, whereas the Ca(OH)₂, chitosan-hydrogel, HA-hydrogel, MTR-hydrogel, and DC-hydrogel groups exhibited ALP levels comparable to those of the control. In contrast, the DAP and TAP groups showed lower ALP activity values than the control group, although this difference was not statistically significant. Meanwhile, the CHX gel and CHX Hgel groups demonstrated significantly lower ALP activity values compared with the other groups. These findings suggest that exposure to certain medicaments may influence the early osteogenic activity of DPSCs, as reflected by changes in ALP levels; however, this interpretation should be considered within the limitations of the in-vitro model and requires further validation in future studies. Figure 3 provides a visual comparison of ALP activity levels among the experimental groups.

**Table 3:** Comparison of ALP activity values in DPSCs after 7 days of exposure to medicament-treated dentine discs

Groups	Mean (SD)	Median (ALP activity)	Minimum	Maximum	*p*-value
Control	99.788 (0.007)	99.781^a^	99.781	99.802	0.003
Chitosan Hgel	99.723 (0.026)	99.738^abce^	99.673	99.759	
DAP	99.526 (0.045)	99.489^b^	99.474	99.616	
TAP	98.386 (1.33)	99.716^c^	95.725	99.716	
Ca(OH)_2_	99.738 (0.021)	99.716^ac^	99.716	99.781	
CHX gel	94.495 (0.597)	93.907^d^	93.89	95.689	
HA Hgel	99.716 (0.012)	99.716^aced^	99.695	99.738	
DC Hgel	99.263 (0.215)	99.311^ecd^	98.868	99.609	
MTR Hgel	99.709 (0.007)	99.716^ac^	99.695	99.716	
CHX Hgel	93.382 (0.135)	93.355^dfc^	93.164	93.628	

Differences between groups were analyzed using the Kruskal–Wallis test (*p* < 0.05), followed by Bonferroni-adjusted post hoc multiple comparisons. Superscript letters represent the results of pairwise comparisons. Groups sharing at least one common superscript letter show no statistically significant difference, whereas groups with no shared letters show a statistically significant difference (*p* < 0.05)

*Abbreviations*: *Chitosan Hgel*, Chitosan Hydrogel; *DAP*, Double Antibiotic Paste; *TAP*, Triple Antibiotic Paste; *Ca(OH)₂*, Calcium Hydroxide Paste; *CHX gel*, Chlorhexidine Gel; *HA Hgel*, Hyaluronic Acid Hydrogel; *DC Hgel*, Diclofenac Hydrogel; *MTR Hgel*, Metronidazole Hydrogel; *CHX Hgel*, Chlorhexidine Hydrogel

## Discussion

In this in vitro study, the antibacterial activities of conventional intracanal medicaments used in regenerative endodontic treatment, such as TAP, DAP, Ca(OH)₂, and CHX gel, were compared with newly developed intracanal medicaments, including chitosan hydrogel, CHX hydrogel, hyaluronic acid hydrogel, metronidazole hydrogel, and diclofenac hydrogel, by evaluating their effects on the *E. faecalis* biofilm and on the viability of dental pulp stem cells (DPSCs).

*Enterococcus faecalis* is a bacterium frequently encountered in resistant endodontic infections and is accepted as the most commonly used microorganism in vitro studies evaluating the antibacterial efficacy of regenerative endodontic treatment (RET) agents [22, 27]. Among the most commonly used agents for canal disinfection in RET protocols are calcium hydroxide (Ca(OH)₂) and various antibiotic combinations. In this context, it is necessary to carefully evaluate both the antimicrobial effectiveness of the applied materials and their biocompatibility with dental stem cells. Previous studies have reported that TAP and DAP, applied at concentrations of 10 mg/ml or higher, can completely eliminate *E. faecalis* biofilm in root canals [36, 37]. In contrast, it has been stated that low concentrations may not show sufficient antibacterial effect on *E. faecalis* [38]. However, it has also been emphasized that high doses of antibiotics may exert cytotoxic effects on dental stem cells, which could negatively affect regenerative processes [39, 40]. Therefore, the ideal antibiotic concentration should be determined in a way that not only maintains antimicrobial efficacy but also ensures cellular biocompatibility.

 In the present study, the dual and triple antibiotic pastes used at a concentration of 2.5 mg/mL resulted in the complete absence of detectable viable *E. faecalis* in CFU analysis after one week of application. In terms of cell viability, it was determined that approximately 69% of dental pulp stem cells remained viable following exposure to the triple antibiotic paste, whereas about 79.5% maintained their viability after exposure to the dual antibiotic paste. This difference is thought to be due to the potential cytotoxic effect of doxycycline, which is included in the triple antibiotic combination. Moreover, the fact that neither of the antibiotic pastes caused a significant change in ALP activity compared to the control group indicates that they did not adversely affect the process of osteogenic differentiation. These findings suggest that the use of antibiotics at low concentrations may provide a balanced approach in terms of both antibacterial efficacy and cellular biocompatibility.

Calcium hydroxide (UltraCal XS, Ultradent, USA) significantly reduced the *E. faecalis* biofilm load; however, complete eradication was not achieved. This finding indicates that the antibacterial efficacy of Ca(OH)₂ is limited and may not be sufficient on its own against resistant microorganisms. This result is also consistent with the adaptive defense mechanisms developed by *E. faecalis* against alkaline pH. In addition, when dental pulp stem cells were applied onto surfaces coated with Ca(OH)₂, 92.6% of the cells remained viable, and no significant decrease in ALP activity was observed compared to the control group. This high biocompatibility demonstrates that the material is safe at the cellular level and supports osteogenic potential in regenerative endodontic treatments; however, the antibacterial efficacy is considered to require enhancement through the addition of other antibacterial agents.

Compared to the calcium hydroxide group, chlorhexidine (CHX) demonstrated a broader antibacterial spectrum. CHX binds to the bacterial cell wall, disrupts membrane integrity, and causes leakage of intracellular contents [41, 42]. Both gel and hydrogel formulations have shown strong antibacterial effects against *E. faecalis* biofilms, with the gel form being particularly more effective against calcium hydroxide–resistant strains [43–45]. CHX containing hydrogels also exhibit a high capacity to disrupt biofilm structure and eliminate residual bacteria, although efficacy varies with formulation type and concentration. Biofilm elimination is more pronounced at concentrations ≥ 1% [46]. However, 2% CHX has been associated with marked cytotoxicity on dental pulp and apical papilla stem cells, resulting in rapid reductions in cell viability [47, 48]. In contrast, low-concentration CHX hydrogels have been shown to maintain cell viability above 70% and to support ALP activity [29, 49].

In the present study, 2% CHX gel (Best Dental, Turkey) produced greater antibacterial effects against *E. faecalis* biofilms compared to calcium hydroxide, significantly reducing biofilm load but not achieving complete eradication. Conversely, the CHX hydrogel completely eliminated the biofilm, indicating that the hydrogel structure enhances the antibacterial efficacy of CHX and is consistent with previous studies. Although cell viability decreased below 50% in both CHX groups, ALP activity remained relatively preserved. This indicates that CHX exposure did not suppress ALP activity to the same extent as metabolic activity under the present experimental conditions.

The antibacterial activity of chitosan against *E. faecalis* varies depending on its concentration and formulation [31, 50]. However, chitosan alone has been reported to show limited efficacy against established biofilms, with improved antibacterial performance achieved through combination therapies or structural modifications [32, 51]. High–molecular weight chitosan may exert stronger antibacterial effects [52], whereas low-concentration formulations have been shown to preserve dental pulp stem cell (DPSC) viability and exhibit favorable cytocompatibility profiles [33]. In addition, hybrid chitosan-based systems have been reported to support cell proliferation, odontogenic differentiation, and ALP activity [30].

 Although chitosan is generally regarded as biocompatible, its in vivo performance may vary according to scaffold architecture and degradation characteristics. Palma et al. demonstrated in an animal model that chitosan-based scaffolds, despite being well tolerated, did not enhance mineralized tissue formation compared with blood clot and were associated with incomplete degradation and persistent inflammatory responses. In some cases, residual scaffold material occupied the canal space, potentially limiting tissue organization and ingrowth [53].

In the present study, a low–molecular weight chitosan hydrogel was selected in consideration of its potential to penetrate dentinal tubules. Consistent with previous reports indicating limited efficacy when used alone, the formulation demonstrated only modest antibacterial activity against *E. faecalis*. Nevertheless, 87.2% of DPSCs remained viable on chitosan-coated surfaces, and ALP activity did not differ from control levels, suggesting that early osteogenic differentiation capacity was not adversely affected. In light of previously reported in vivo findings and the limited biofilm control observed when chitosan is applied as a single agent, not only cytocompatibility but also antibacterial performance and biodegradation characteristics should be carefully considered when evaluating its clinical applicability. Within this context, chitosan hydrogels may hold potential as biologically compatible carrier matrices in regenerative endodontics and could be rendered more effective through optimized formulation strategies.

Hyaluronic acid (HA), another natural polymer, is known for its capacity to support cell proliferation, migration, and tissue healing [54]. HA-based hydrogels have been incorporated into regenerative endodontic strategies, including growth factor–releasing systems designed to enhance pulp regeneration [55]. Furthermore, experimental studies have demonstrated that HA hydrogels promote the viability, spreading, and metabolic activity of DPSCs and SCAPs, supporting their role in cell-mediated regenerative processes [56, 57]. In parallel, contemporary developments in endodontic drug delivery highlight the importance of systems that integrate antimicrobial control with stem cell compatibility through controlled release mechanisms [58]. Within this broader therapeutic framework, HA hydrogels may also be positioned as biologically compatible matrices with potential carrier functionality, aligning with established stem cell–based pulp–dentin regeneration models that serve as biological reference systems in regenerative research [59].

 In the present study, the HA hydrogel demonstrated limited antibacterial activity against *E. faecalis*. However, 79.8% of DPSCs remained viable following exposure to HA-treated surfaces, and ALP activity did not differ from control levels, indicating preservation of early osteogenic differentiation capacity. Collectively, these findings indicate that although HA exhibits modest intrinsic antibacterial performance under the tested conditions, it maintains a stable biocompatibility profile and preserves osteogenic potential, supporting its consideration as a biologically suitable carrier matrix in regenerative endodontic applications. 

Diclofenac (DC), a widely used nonsteroidal anti-inflammatory drug (NSAID), has recently attracted interest in regenerative endodontics for its antimicrobial potential. Studies indicate that DC can inhibit both Gram-positive and Gram-negative bacteria, including resistant strains such as *E. faecalis*, in a concentration-dependent manner [60, 61]. 

Its antibacterial activity extends beyond simple membrane disruption, affecting key bacterial processes such as membrane permeability and intracellular homeostasis, contributing to disruption of biofilm structure and function [60, 62]. In addition, diclofenac and other NSAIDs may act through cyclooxygenase-independent mechanisms, including modulation of efflux pumps and interference with bacterial metabolism, further enhancing susceptibility within biofilm communities [60, 62]. 

At the same time, DC can exhibit dose-dependent cytotoxicity toward mammalian cells, including human dental pulp stem cells (DPSCs) [63]. In vitro evidence shows that exposure can reduce DPSC viability and alter apoptosis-related gene expression, highlighting the need to optimize the therapeutic dosage when applied in regenerative protocols [63, 64].

In the present study, the DC hydrogel completely eradicated *E. faecalis* biofilms, demonstrating strong antibacterial efficacy. Furthermore, DPSCs cultured on DC-treated surfaces preserved 79.6% viability, and no adverse changes in ALP activity were observed. Importantly, the concentration applied in this study appeared to remain within a biologically acceptable range, balancing effective biofilm elimination with preservation of stem cell viability. These findings suggest that, under controlled in vitro conditions, diclofenac can achieve effective biofilm eradication while maintaining acceptable biocompatibility and preservation of osteogenic potential. Nevertheless, further studies are required to evaluate long-term cellular responses and to confirm the safety profile of diclofenac in regenerative endodontic applications.

Metronidazole (MTR) is well known for its efficacy against anaerobic bacteria but exhibits limited activity against facultative anaerobes such as *E. faecalis* [65]. To enhance its antibacterial performance, combination with other antimicrobial agents or incorporation into controlled-release systems has been proposed [45, 66].

In the present investigation, the MTR hydrogel failed to achieve complete eradication of *E. faecalis* biofilms and demonstrated only limited antibacterial efficacy. This finding is consistent with previous studies reporting insufficient effects of MTR when used alone. Despite the hydrogel formulation, the restricted efficacy observed suggests that optimized carrier systems or combination with additional antimicrobial agents are necessary for MTR to be effective in biofilm environments. Considering the presence of multispecies communities and complex biofilm structures in clinical situations, the limitations of MTR as a stand-alone agent become even more evident. Nevertheless, 79.6% of DPSC viability was preserved, and ALP activity remained high, confirming the low cytotoxicity of MTR and its lack of inhibitory effects on osteogenic potential. These characteristics suggest that, despite its limited antibacterial activity, MTR may serve as a safe and supportive component within combined treatment strategies for regenerative endodontic applications.

Alkaline phosphatase (ALP) activity is widely accepted as an early marker of osteogenic differentiation; however, it does not necessarily reflect long-term mineralization capacity. Experimental studies conducted under inflammatory conditions have shown that osteogenic markers, including ALP, may be downregulated even when early cell viability is relatively preserved, indicating that cell survival and differentiation potential do not always progress in parallel [67]. In addition, biomaterials and carrier systems have been reported to modulate early osteogenic responses through cell–material interactions rather than acting solely as passive delivery platforms [68]. Within this context, the preserved ALP activity observed in several hydrogel groups in the present study may not be attributable only to low cytotoxicity, but also to material-related biological interactions influencing early differentiation processes. Therefore, ALP findings should be interpreted together with cell viability and antibacterial efficacy outcomes rather than as a standalone indicator of regenerative potential [67, 68].

As this study was conducted under in vitro conditions, the biological findings should be regarded as preliminary and should not be directly translated into clinical practice at this stage. Although canal preparation was standardized and a dentine powder model was used, these experimental conditions cannot fully replicate the complexity of the clinical microenvironment. In addition, the conclusions represent statistical inferences derived from the available sample, and the limited sample size may restrict the generalizability of the findings. Therefore, the clinical applicability of these results should be interpreted with caution and requires validation through well-designed in vivo and long-term studies.

## Conclusions

In this in-vitro study, hydrogel-based systems demonstrated a balanced profile between biofilm control and stem-cell biocompatibility. The diclofenac-loaded hydrogel achieved TAP/DAP-level biofilm reduction while preserving cell viability and ALP activity, suggesting a clinically relevant potential as an antibiotic-free disinfection alternative in regenerative endodontics. The CHX hydrogel formulation showed higher antibacterial performance than CHX gel, whereas the hyaluronic-acid hydrogel maintained high biocompatibility and may serve as a biofunctional carrier platform.

## Data Availability

The data that support the findings of this study are available from the corresponding author upon reasonable request.

## Abbreviations

ALP: Alkaline Phosphatase
AAE: American Association of Endodontists
BHI: Brain Heart Infusion
CFU: Colony-Forming Unit
CHX: Chlorhexidine
CHX gel: Chlorhexidine Gel
CHX Hgel: Chlorhexidine Hydrogel
CLSM: Confocal Laser Scanning Microscopy
DAP: Double Antibiotic Paste
DC: Diclofenac
DC Hgel: Diclofenac Hydrogel
DMEM: Dulbecco’s Modified Eagle Medium
DPSC(s): Dental Pulp Stem Cell(s)
EDTA: Ethylenediaminetetraacetic Acid
FBS: Fetal Bovine Serum
HA: Hyaluronic Acid
HA Hgel: Hyaluronic Acid Hydrogel
Hgel: Hydrogel
MTR: Metronidazole
MTR Hgel: Metronidazole Hydrogel
NaOCl: Sodium Hypochlorite
NSAID: Non-Steroidal Anti-Inflammatory Drug
PBS: Phosphate Buffered Saline
REP(s) / REPs: Regenerative Endodontic Procedure(s)
RET: Regenerative Endodontic Treatment
SCAP: Stem Cells of the Apical Papilla
SEM: Scanning Electron Microscopy / Microscope
TAP: Triple Antibiotic Paste
TSA: Tryptic Soy Agar
